# Cancer surgical outcome study in Ethiopia: A 7-day multicenter prospective observational cohort study

**DOI:** 10.1371/journal.pone.0354980

**Published:** 2026-07-29

**Authors:** Atalel Fentahun Awedew, Yonatan Abie Tsegaye, Addisu Assfaw Ayen, Fisiha Guade Tesifaw, Bedemariam Tadesse Amsalu, Amsalu Molla Getahun

**Affiliations:** 1 Department of Surgery, SOM, Debre Tabor University, Debre Tabor, Ethiopia; 2 Department of Orthopedics and Trauma Surgery, SOM, Debre Tabor University, Debre Tabor, Ethiopia; 3 Department of Internal Medicine, SOM, Debre Tabor University, Debre Tabor, Ethiopia; 4 Department of Surgery, SOM, Bahir Dar University, Bahir Dar, Ethiopia; 5 Department of Surgery, CHS, University of Gondar, Gondar, Ethiopia; Jimma University College of Public Health and Medical Sciences, ETHIOPIA

## Abstract

**Background:**

Safe surgery is a fundamental quality indicator within the global surgery framework. Postoperative complications remain a leading global cause of disability, mortality, and economic loss, with a disproportionate impact on low- and middle-income countries (LMICs).

**Objective:**

This study aims to generate robust epidemiological data on postoperative outcomes of cancer surgery in Ethiopia.

**Methods:**

This study employed a 7-day national observational prospective cohort design. The study enrolled adult patients aged 18 years and older who underwent either elective or non-elective cancer surgery in hospitals across Ethiopia. Statistical analysis included descriptive statistics, chi-square tests for categorical variables, and logistic regression models to identify risk factors for postoperative complications. Outcome measures 7^th^ day postoperative mortality and complications. Statistical significance was set at p < 0.05.

**Results:**

A total of 265 cancer surgeries were performed across 46 hospitals (overall surgeries = 4412). The mean patient age was 46.6 years (SD = 14.9). The majority of patients were classified as low-risk America Society of Anesthesiologists (ASA) physical status classification: ASA I (106/265, 40%) and ASA II (133/265, 50.2%) and low Eastern Cooperative Oncology Group (ECOG) performance status: ECOG 0 (112/265, 42.3%) and ECOG 1 (98/265, 37%). Colorectal cancer surgery was the most common type (38/265, 14.5%), while laparoscopic surgery was performed in only 3/265 cases (1.1%). Postoperative complications developed in 84 of 265 patients (31.7%) with leading complication was superficial surgical site infection 47/265(17.8%). Emergency surgery (AOR = 3.1, 95% CI: 1.1–8.4, p = 0.003), comorbidity (AOR = 2.0, 95% CI: 1.1–3.7, p = 0.025), and an ECOG performance status of III (AOR = 9.5, 95% CI: 1.5–61.1, p = 0.02) were statistically significantly associated with 7^th^ day postoperative complications. The overall 7-day mortality rate after cancer surgery was 5/265 (1.9%).

**Conclusion:**

Despite the relatively young age of patients, their low ASA physical status scores, and good ECOG performance, a significant proportion experienced adverse outcomes following cancer surgery: one in three patients developed postoperative complications, one in nine required reoperations, and one in 53 died. These findings suggest a critical need for evidence-based interventions to strengthen the underlying infrastructure and care processes within the surgical system which are essential to achieving safe, effective, and high-quality surgical care for cancer patients in Ethiopia.

## Introduction

Cancer remains a substantial global health challenge, contributing significantly to mortality, morbidity, and disability worldwide [1–3]. Cancer ranks as the second leading cause of global deaths and disability worldwide, with the burden falling disproportionately on low- and middle-income countries (LMICs), including Ethiopia, whose healthcare systems often face considerable resource constraints [1–3].

Surgical health care plays a crucial role across the cancer continuum, encompassing prevention, control, diagnosis, treatment, and rehabilitation [4]. Access to safe, affordable, and equitable surgical services integrate to achieve universal health coverage, Sustainable Development Goals and national cancer control plans [5]. Cancer surgery performs for a wide array of purposes, including curative, palliative, diagnosis, and reconstruction interventions [6]. Approximately 80% of all cancers necessitate some form of surgical management [6], resulting in estimated global need for 32 million cancer-related operations annually as of 2015, a figure projected to increase to 45 million by 2030, emphasizing the escalating demand for surgical services in cancer care [6]. Furthermore, the most recent modeling data from 2021 projects a substantial 52% increase in global surgical cancer cases between 2018 and 2040 [7]. This projection indicates an approximate need for an additional 5 million procedures for cancer-related indications in 2040 compared to 2018, highlighting the urgency of expanding surgical capacity and resources [7].

Providing quality cancer surgery is a complex undertaking requiring substantial human, financial, and technological resources [6]. This often necessitates access to advanced imaging, pathology services, and adjunct oncologic therapies, such as chemotherapy, radiotherapy, and immunotherapy [8]. Due to the significant resource demands, access to quality cancer surgery is markedly disparate: less than 5% of low- and middle-income countries (LMICs) are able to provide adequate, affordable, equitable, and safe cancer surgical care, while more than 95% of high-income countries (HICs) offer readily available access to such services [8]. This stark disparity highlights the profound inequalities in global cancer care [8].

Surgical care has historically been neglected within national public health agendas in Ethiopia, a common challenge across many low- and middle-income countries (LMICs) [9]. The Lancet Commission on Global Surgery has, however, spurred a positive shift in awareness and policy focus, including in Ethiopia. The country developed a national surgical policy, known as “Saving Lives through Safe Surgery” (SaLTS), in 2015 [10], which primarily aimed to institutional capacity building which able to perform basic essential surgery, surgical workforce development and Task-shifting/Task sharing modeling to achieve safe and equitable surgical care. Despite these positive developments, the policy framework has yet to adequately address the specific challenges of cancer surgery, resulting in significant gaps in surgical outcomes for cancer patients in Ethiopia. Therefore, this study aims to address the evidence gap on cancer surgical outcomes in Ethiopia, generating actionable data to inform evidence-based policy, advance cancer surgical research, and providing essential insights for policymakers, researchers, and healthcare professionals.

## Methodology

### Study design

We conducted a national, prospective, observational cohort study across 46 hospitals in all regions of the country. Each participating hospital selected one month from January to April 2024 as their data collection period. During this month, all consecutive patients admitted for elective or non-elective inpatient cancer surgery -with a planned overnight stay -were enrolled. Each enrolled patient was then followed for 7 days after their surgery to monitor postoperative outcomes. Patients undergoing planned day cancer surgery, pediatrics (age < 18 years), or radiological procedures not requiring anaesthesia were excluded.

### Data collection

The data collection tool was designed based on previous international surgical outcome studies. It captured key variables across the preoperative, intraoperative, and postoperative phases of surgical care. Data were collected using well-validated data collection tool used for the Africa Surgical Outcome Study (ASOS) [11] and International Surgical Outcome Study (ISOS) [12]. The outcome measure included 7^th^ day in-hospital mortality, post-operative complication and prevalence of critical illness. The data were collected by surgeons, medical interns, residents, and anesthetists. Detailed tracking of complications severity, such as infectious complication, cardiovascular complication, and other complications were assessed. Data collection tools provided at **Supplement file 1.**

### Data analysis

All collected data underwent supervisor review and systematic validation. A designated supervisor examined each completed questionnaire for accuracy, consistency, and completeness, cross-referencing assigned participant codes throughout the process. Any discrepancies or missing entries were resolved through re-verification with the original data source prior to entry into the study database. Data analysis was performed using SPSS software, version 25. Categorical variables were summarized using frequencies and percentages, and comparisons between groups were conducted using the chi-square test. The normality of continuous variables was assessed using the Shapiro-Wilk test. Normally distributed data were described using means and standard deviations, while skewed data were characterized using medians and interquartile ranges. Prior to multivariable analysis, a multicollinearity test was performed to evaluate potential correlations between categorical, continuous, and binary variables. Multicollinearity was assessed using the variance inflation factor (VIF) and tolerance values. Variables exhibiting a VIF below 5 and a tolerance above 0.1 were considered sufficiently independent and were subsequently included in the multivariable binary logistic regression analysis. Conversely, variables with a VIF score ranging from 5 to 10 or higher, and a tolerance value below 0.1, were excluded from the final model to avoid issues arising from multicollinearity. To identify potential predictors, a univariate binary logistic regression analysis was initially performed to examine factors associated 7^th^ day postoperative complications. Variables demonstrating a p-value of 0.2 or less in the bivariate analysis were then entered into a multivariable binary logistic regression model. This model was used to assess the independent associations between the various factors and the outcomes of 7^th^ -day postoperative complications. The results of the logistic regression analysis are reported as adjusted odds ratios (OR) with corresponding 95% confidence intervals. A p-value less than 0.05 was used as the threshold for statistical significance.

### Ethical consideration

This study was part of the Ethiopian Surgical Outcome Study (EthioSOS). Ethical approval was obtained from the Institutional Review Board of the Armauer Hansen Research Institute (AHRI) under Protocol No. PO-64–23. Informed written consent to participate was obtained from all of the participants in the study. The study also adhered with the principle and recommendation of Helsinki Declaration.

## Results

A total of 265 cancer surgeries were performed across 46 hospitals (overall surgery = 4412), with a mean patient age of 46.6 years (SD = 14.9) with slight female predominance (154/265, 58.1%). The majority of patients were classified as low risk according to the ASA physical status classification: ASA I (106/265, 40%) and ASA II (133/265, 50.2%). Similarly, most patients had a low ECOG performance status: ECOG 0 (112/265, 42.3%) and ECOG 1 (98/265, 37%). Comorbidities were present in 96 of 265 patients (36·2%). Colorectal cancer surgery (38/265, 14.5%) and breast cancer surgery (27/265, 10.2%) were the most common procedures performed. Most surgeries were elective (160/265, 60.4%) and classified as major (205/265, 77.4%). Underutilization of the WHO Surgical Safety Checklist was observed in 42/265 (15.8%) of cases, and laparoscopic surgery was performed in only 3/265 (1.1%) of cases. Approximately 43/265 (16.2%) of patients required immediate critical care admission (Table 1)

**Table 1:** Baseline characteristics of the Cancer Surgical Outcomes Study in Ethiopia.

Age	Mean + SD	46.6 + 14.9	
Sex	Female	154	58.1
	Male	111	41.9
ASA	I	106	40.0
	II	133	50.2
	III	26	9.8
Comorbidity	No	169	63.8
	Yes	96	36.2
ECOG	0	112	42.3
	I	98	37.0
	II	46	17.4
	III	9	3.4
Cancer type	Colorectal	38	14.3
	Renal CA	11	4.2
	Ortho cancer	15	5.7
	Lung	8	3.0
	Skin cancer	7	2.6
	Esophagus	11	4.2
	Stomach	14	5.3
	Parathyroid	1	.4
	Gynecological ca	19	7.2
	ENT	16	6.0
	HPB	20	7.5
	Breast	27	10.2
	Thyroid	24	9.1
	Bladder	22	8.3
	Small bowel ca	7	2.6
	Neuro-cancer	20	7.5
	Adrenal	3	1.1
	Penile ca	2	.8
Timing of surgery	Elective	160	60.4
	Emergency	23	8.7
	Urgent	82	30.9
Severity of surgery	Intermediate/minor	60	22.6
	Major	205	77.4
Laparoscopy surgery	No	262	98.9
	Yes	3	1.1
WHO safety checklist	No	42	15.8
	Yes	223	84.2
Critical admission	No	222	83.8
	Yes	43	16.2

### Cancer surgical outcome

Postoperative complications developed in 84 of 265 patients (31.7%). According to the Clavien-Dindo classification, 29 of 265 patients (10.9%) experienced Grade 1 complications, and 30 of 265 patients (11.3%) experienced Grade 2 complications. Grade III or higher postoperative complications, requiring either radiological or open surgical intervention, developed in 29/265 patients (11.1%). The most frequent complications were superficial surgical site infection in 47 of 265 patients (17·8%), deep surgical site infection in 18 of 265 (6·8%), and bloodstream infection in 11 of 265 (4·2%). The overall 7-day postoperative mortality rate was 5 of 265 patients (1·9%). (Table 2 and 3).

**Table 2:** Postoperative outcomes of Cancer surgical outcomes study in Ethiopia.

	Response	Frequency	%
Postoperative complications	No	181	68.3
	Yes	84	31.7
Clavien-Dindo classification	Grade 1	29	10.9
	Grade 2	30	11.3
	Grade IIIA	11	4.2
	Grade IIIB	4	1.5
	Grade IV A	8	3.0
	Grade IV B	2	.8
Reoperation (n = 29)	Radiological	7	11.1%
	Open surgical	22	
7^th^ day	Died	5	1.9
	Discharged	165	62.3
	Still admitted – in hospital ward	91	34.3
	Still admitted – in the critical care unit	4	1.5

**Table 3:** Type of surgical complications after cancer surgery in Ethiopia.

Complication	Mild	Moderate	Sever	Total
Superficial surgical site infection	22(8.3%)	19(7.2%)	5(1.9)	47(17.8%)
Deep surgical site infection	9(3.4%	4(1.5%)	5(1.9%)	18(6.8%)
Pneumonia	1(0.4%0	8(3%)	2(0.8%)	11(4.2%)
Bloodstream infection	6(2.3%)	1(0.4%)	4(1.5)	11(4.2%)
Urinary Tract Infection	4(1.5)	4(1.5)	1(0.4%0	9(3.4%)
Arrhythmia	1(0.4%0		2(0.8%)	3(1.2%)
Acute kidney Injury	6(2.3%)	1(0.4%)	2(0.8%)	9(3.4%)
Anastomosis Leak			5(1.9)	5(1.9)

Several factors are associated with postoperative complications following cancer surgery. After adjusting for confounders, the following factors were statistically significantly associated with postoperative complications: emergency surgery (AOR = 3.1, 95% CI: 1.1–8.4, p = 0.003), comorbidity (AOR = 2.0, 95% CI: 1.1–3.7, p = 0.025), an ECOG performance status of III (AOR = 9.5, 95% CI: 1.5–61.1, p = 0.02), and age older than 45 years (AOR = 2.2, 95% CI: 1.2–4.1, p = 0.008) (Table 4)

**Table 4:** Univariable and multivariable binary logistic regression analysis to identified factors associated with postoperative complications after cancer surgery in Ethiopia.

Variables	Response	Yes	No	COR	AOR	P
Age	below 45	30	99	1	1	
	above 45	54	82	2.2(1.3,3.7)	2.2(1.2,4.1)	0.008*
ASA	I	22	84	1	1	
	II	47	86	2.1(1.2,3.8)	1.5(0.8,2.9)	0.25
	III	15	11	5.2(2.1,12.9)	2.5(0.8, 7.2)	0.9
Time of surgery	Elective	40	120	1	1	
	Emergency	13	10	3.9(1.6,9.6)	3.1(1.1,8.4)	0.03*
	Urgent	31	51	1.8(1.0,3.2)	2.5(1.3,4.7)	0.007*
Severity surgery	Intermediate/minor	11	49	1	1	
	Major	73	132	2.5(1.2,5.1)	2.8(1.4,6.3)	0.013*
Comorbidity	No	39	130	1	1	
	Yes	45	51	2.9(1.7,5.0)	2.0(1.1,3.7)	0.025*
ECOG	0	24	88	1	1	
	I	31	67	1.7(0.9,3.2)	1.2(0.6,2.4)	0.6
	II	22	24	3.4(1.6,7.0)	2.6(1.2,6.0)	0.022*
	III	7	2	12.8, (2.5,65.8)	9.5(1.5,61.1)	0.02*

## Discussion

This study reveals the substantial burden of cancer surgery in Ethiopia, with approximately one in 17 surgical procedures performed in the nationally attributable to cancer. The leading cancer types requiring operative intervention were colorectal, breast, hepatobiliary and pancreatic, and neurological malignancies. Notably, one in three patients experienced postoperative complications, one in nine required reoperations, and one in 53 died within seven days of cancer surgery.

The postoperative complication rate observed in our study- approximately one in three patients – was three times in the Africa Surgical Outcome Study (ASOS) [11] and the International Surgical Outcome Study [12], which reported the overall complication approximately 18% and 17% respectively [11,12]. This discrepancy is not easily explained by patients baseline sociodemographic and clinical characteristics; our patients were young, low ASA, good ECOG and low comorbidity rate- parameters associated with favorable operative risks. The high complication rate observed in our study implicates systemic and structure factors inherent from the surgical care delivery system, rather that patient level physiology alone. The delay diagnosis, advanced presentation, poor preoperative optimization, neoadjuvant optimization, intraoperative techniques and postoperative monitoring are likely contributors. These findings are consistent with evidence from the GlobalSurg Collaborative, which demonstrated that complication profiles in LMICs are disproportionately shaped by hospital and country-level factors, independent of individual patient risk [13].

The Lancet Commission on Global Surgery identified the perioperative mortality rate (POMR) as a cardinal indicator of surgical system strength [5]. Ethiopian Ministry of Health adopted the lancet recommendations and planning a national target of less than 2% by 2025 [14]. The seven-day POMR of approximately 2% in the present study was broadly consistent with rates reported in the African Surgical Outcomes Study (ASOS: 2.1%) [11] and the International Surgical Outcomes Study (ISOS: 1.5%) [12], nominally meeting the national benchmark However, this figure warrants careful interpretation: our cohort was markedly younger and had substantially lower physiological risk, as reflected by lower ASA and ECOG scores, compared with ASOS, ISOS, and high-income country populations. A 2% POMR in a younger, functionally preserved cohort likely represents a disproportionately higher risk-adjusted mortality than the equivalent rate observed in older, more comorbid populations elsewhere. Meeting the national target should therefore not be equated with optimal surgical safety, and risk-adjusted benchmarking remains essential for driving meaningful quality improvement in Ethiopian cancer surgical centres.

We also found that reoperation rate was approximately one in nine patients, which is higher from previous studies [15]. Unplanned return to operation room is a widely accepted surgical quality indicators, reflecting multiple stage failure during surgery delivery from preoperative decision making to post operative surveillance. Reoperations carry compounded risks: each additional anaesthetic exposure and procedure substantially increases both mortality risk and length of stay, placing additional demands on already-strained theatre capacity, blood bank resources, and intensive care facilities. Reducing avoidable reoperations must therefore be considered a strategic priority for quality improvement programmes in Ethiopian surgical centres.

Evidence from large-scale, multicentre international studies spanning 82 countries demonstrates that adverse postoperative outcomes following cancer surgery arise from a dual burden of patient-specific and health-system determinants [13]. Approximately 60% of postoperative complications are attributable to patient-level factors- including delayed presentation, advanced disease stage, tumour histology, and functional reserve- while the remaining 40% are independently linked to hospital and country-level factors, encompassing diagnostic capability, perioperative care quality, and human resource capacity, as quantified by the Socio-Demographic Index (SDI) [13]. Of particular concern, inconsistent availability of postoperative care facilities in LMICs has been associated with an excess of seven to ten deaths per 100 major complications [13]- a preventable toll that represents a measurable indicator of health system inequity. Investment in high-dependency and intensive care capacity must therefore be recognised as a core, rather than ancillary, component of Ethiopia’s surgical expansion agenda.

The Ethiopian Ministry of Health’s Saving Lives Through Safe Surgery (SaLTS) initiative represents an important national commitment to expanding surgical access and improving operative quality, primarily through the strengthening of essential service delivery capacity, facility infrastructure, and surgical workforce development [16]. Our findings, however, reveal a significant performance gap relative to the SaLTS II 2025 targets- specifically, a surgical site infection rate below 5%, underscoring the distance that remains between policy ambition and clinical reality. Robust and sustained financing of surgical infrastructure and human resource capacity constitutes the foundational prerequisite for delivering safe, equitable, and affordable surgical care, and is integral to achieving universal health coverage (UHC) and the Sustainable Development Goals (SDGs).In this context, Ethiopia’s public health expenditure -which remains below 5% of the national budget and falls substantially short of the 15% benchmark enshrined in the Abuja Declaration -represents a critical structural impediment to surgical system strengthening, a challenge shared by many low- and middle-income countries. Without a substantial and sustained reorientation of public investment toward surgical care, encompassing infrastructure, workforce capacity, essential medicines, and quality improvement systems, progress in surgical oncology will remain geographically inequitable, systemically fragile, and inadequate to address the growing population burden of cancer.

The Lancet Commission on Global Surgery has proposed the safe and equitable surgical cares are a cardinal national indicator for evaluating surgical system strength [5]. Building on this foundation, the Lancet Global Health Commission on High-Quality Health Systems articulated a tripartite framework for achieving safe, affordable, and timely surgical care, comprising essential foundations -encompassing infrastructure, workforce, and financing – robust care processes, including quality assurance, patient safety systems, and health data infrastructure, and demonstrable quality impact as the measurable expression of system performance [17]. Translating this framework into actionable reform within the Ethiopian context demands a coherent and sustained policy response: national cancer surgery planning must be explicitly aligned with universal health coverage (UHC) principles; surgical care must be formally integrated into the essential health benefits package to ensure equitable access across socioeconomic strata; and routine surgical audit, morbidity and mortality review, and structured quality improvement cycles must be institutionalized as standard practice across all levels of the health system. Collectively, these measures represent the minimum programmatic architecture required to close the prevailing gap between surgical policy aspiration and clinical reality, and to deliver safe, equitable, and affordable cancer surgical care to the Ethiopian population.

There are some limitations inherent to this study that warrant careful consideration. The seven-day observational window represents a constrained follow-up period that likely underestimates the true incidence of postoperative mortality and morbidity, given that a substantial proportion of infectious, nutritional, and wound-related complications characteristically manifest beyond this timeframe, thereby potentially obscuring the full burden of adverse surgical outcomes. Furthermore, although the multicenter design enhances generalizability across diverse healthcare settings in Ethiopia, unmeasured institutional variability in infrastructure, surgical technique, perioperative protocols, and postoperative care standards may introduce heterogeneity that limits direct comparability across participating centers. Additionally, as with all observational cohort studies, the possibility of residual confounding from unmeasured clinical, socioeconomic, and pathological variables cannot be entirely excluded, which may influence the magnitude and direction of observed associations. Therefore, future research should address longer-term follow-up extending beyond the immediate seven-day postoperative period to capture late complications and survival outcomes. Additionally, subsequent investigations should also examine hospital-level and health-system-level determinants, including workforce training, infrastructural capacity, and perioperative protocols, that may account for the observed variations in surgical outcomes.

Conclusion: Our findings indicate that postoperative complication rates were significantly higher, nearly three, times compared to those reported in high-income countries. This is particularly concerning given that our patient population was relatively young and had low ASA class and ECOG performance scores, typically indicators of lower risk. These results strongly suggest that incompetent in care processing system and the foundational elements of surgical service delivery in Ethiopia, significantly compromise the quality dimension of surgical care

## Supporting information

S1 File Data collection tool. (DOCX)

S2 File Cancer outcome in Ethiopia.xlsx (analyzed Data). (XLSX)

## Abbreviations

ASOS: African Surgical Outcomes Study
AOR: Adjusted Odd Ratio
ASA: American Society of Anesthesiologists
ECOG: Eastern Cooperative Oncology Group
HICs: High, income countries
ISOS: International Surgical outcome study
LMICs: Low, and middle, income countries (LMICs)
POMR: Perioperative mortality rate (POMR)
SaLTS: Saving Lives through Safe Surgery
SD: Standardized deviation
SPSS: Statistical Package for the Social Sciences
UHC: Universal health coverage
VIF: Variance inflation factor
WHO: World Health Organization
