# Supplementary material for: Cancer surgical outcome study in Ethiopia: A 7-day multicenter prospective observational cohort study
Source: PLoS One. 2026 Jul 29;21(7):e0354980. doi: 10.1371/journal.pone.0354980 (PMC13419167; doi:10.1371/journal.pone.0354980)
Supplement: S1 File — (DOCX) [file pone.0354980.s001.docx]

**Supplement file 1: Data collection Tool**

**Name of hospital…………………**

**ID…………………………….**

**Age years**

**Gender ⬜ M ⬜ F**

**Current smoker ⬜ Y ⬜ N**

**Alcohol drunker ⬜ Y ⬜ N**

**Date of Decision Note (Granted admission to liaison office) …………………….**

**Date of hospital admission……………………………………..**

**Date of Surgery……………………………………………………**

**ASA ⬜ I ⬜ II ⬜ III ⬜ IV**

**Current Definitions and ASA-Approved Examples**

| **ASA PS Classification** | **Definition** | **Adult Examples, Including, but not Limited to:** | **Pediatric Examples, Including but not Limited to:** | **Obstetric Examples, Including but not Limited to:** |
| --- | --- | --- | --- | --- |
| **ASA I** | **A normal healthy patient** | **Healthy, non-smoking, no or minimal alcohol use** | **Healthy (no acute or chronic disease), normal BMI percentile for age** |  |
| **ASA II** | **A patient with mild systemic disease** | **Mild diseases only without substantive functional limitations. Current smoker, social alcohol drinker, pregnancy, obesity (30<BMI<40), well-controlled DM/HTN, mild lung disease** | **Asymptomatic congenital cardiac disease, well controlled dysrhythmias, asthma without exacerbation, well controlled epilepsy, non-insulin dependent diabetes mellitus, abnormal BMI percentile for age, mild/moderate OSA, oncologic state in remission, autism with mild limitations** | **Normal pregnancy*, well controlled gestational HTN, controlled preeclampsia without severe features, diet-controlled gestational DM.** |
| **ASA III** | **A patient with severe systemic disease** | **Substantive functional limitations; One or more moderate to severe diseases. Poorly controlled DM or HTN, COPD, morbid obesity (BMI ≥40), active hepatitis, alcohol dependence or abuse, implanted pacemaker, moderate reduction of ejection fraction, ESRD undergoing regularly scheduled dialysis, history (>3 months) of MI, CVA, TIA, or CAD/stents.** | **Uncorrected stable congenital cardiac abnormality, asthma with exacerbation, poorly controlled epilepsy, insulin dependent diabetes mellitus, morbid obesity, malnutrition, severe OSA, oncologic state, renal failure, muscular dystrophy, cystic fibrosis, history of organ transplantation, brain/spinal cord malformation, symptomatic hydrocephalus, premature infant PCA <60 weeks, autism with severe limitations, metabolic disease, difficult airway, long term parenteral nutrition. Full term infants <6 weeks of age.** | **Preeclampsia with severe features, gestational DM with complications or high insulin requirements, a thrombophilic disease requiring anticoagulation.** |
| **ASA IV** | **A patient with severe systemic disease that is a constant threat to life** | **Recent (<3 months) MI, CVA, TIA or CAD/stents, ongoing cardiac ischemia or severe valve dysfunction, severe reduction of ejection fraction, shock, sepsis, DIC, ARD or ESRD not undergoing regularly scheduled dialysis** | **Symptomatic congenital cardiac abnormality, congestive heart failure, active sequelae of prematurity, acute hypoxic-ischemic encephalopathy, shock, sepsis, disseminated intravascular coagulation, automatic implantable cardioverter-defibrillator, ventilator dependence, endocrinopathy, severe trauma, severe respiratory distress, advanced oncologic state.** | **Preeclampsia with severe features complicated by HELLP or other adverse event, peripartum cardiomyopathy with EF <40, uncorrected/decompensated heart disease, acquired or congenital.** |
| **ASA V** | **A moribund patient who is not expected to survive without the operation** | **Ruptured abdominal/thoracic aneurysm, massive trauma, intracranial bleed with mass effect, ischemic bowel in the face of significant cardiac pathology or multiple organ/system dysfunction** | **Massive trauma, intracranial hemorrhage with mass effect, patient requiring ECMO, respiratory failure or arrest, malignant hypertension, decompensated congestive heart failure, hepatic encephalopathy, ischemic bowel or multiple organ/system dysfunction.** | **Uterine rupture.** |
| **ASA VI** | **A declared brain-dead patient whose organs are being removed for donor purposes** |  |  |  |

**Eastern Cooperative Oncology Group (ECOG ⬜ 0 ⬜ I ⬜ II ⬜ III ⬜ IV ⬜ V**

| **GRADE** | **ECOG PERFORMANCE STATUS** |
| --- | --- |
| **0** | **Fully active, able to carry on all pre-disease performance without restriction** |
| **1** | **Restricted in physically strenuous activity but ambulatory and able to carry out work of a light or sedentary nature, e.g., light house work, office work** |
| **2** | **Ambulatory and capable of all selfcare but unable to carry out any work activities; up and about more than 50% of waking hours** |
| **3** | **Capable of only limited selfcare; confined to bed or chair more than 50% of waking hours** |
| **4** | **Completely disabled; cannot carry on any selfcare; totally confined to bed or chair** |
|  |  |

**Comorbidity chronic Disease (*tick all that apply*):**

**⬜Congestive heart failure⬜Yes ⬜None**

**⬜Myocardial infarction⬜Yes ⬜None**

**⬜Peripheral vascular disease⬜Yes ⬜None**

**⬜Hemiplegia⬜Yes ⬜None**

**⬜ Ischemic heart disease⬜Yes ⬜None**

**⬜ HIV/AIDS⬜Yes ⬜None**

**⬜ Hypertension ⬜Yes ⬜None**

**⬜Dementia⬜Yes ⬜None**

**⬜Peptic ulcer disease**

**⬜Connective tissue disease⬜Yes ⬜None**

**⬜ Chronic Kidney disease⬜Yes ⬜None**

**⬜ Diabetes Mellitus ⬜None/Diet controlled ⬜Uncomplicated ⬜End-organ damage**

**⬜ Liver disease(Definition=Severe = cirrhosis and portal hypertension with variceal bleeding history, moderate = cirrhosis and portal hypertension but no variceal bleeding history, mild = chronic hepatitis (or cirrhosis without portal hypertension) ⬜None ⬜Mild ⬜Moderate to sever**

**⬜ Solid tumor ⬜ None ⬜ Localized ⬜ Metastasis**

**⬜ Leukemia⬜Yes ⬜None**

**⬜ Lymphoma⬜Yes ⬜None**

**⬜ Stroke/TIA ⬜Yes ⬜None**

**⬜ COPD / Asthma ⬜Yes ⬜None**

**⬜ Other (Mention …)**

**Most recent blood results (no more than 28 days before surgery):**

**Haemoglobin . g/L Leucocytes . x10^9^/L**

**Sodium mmol/L Creatinine . µmol/L**

**AST ⬜ ALT⬜ ALP ⬜**

**CEA mmol/L**

**CA-19**

**AFP**

**HBV…..Postive/ Negative HCV….Postive/Negative**

4

0

1

2

M

0

D

D

m

m

H

H

**Anaesthesia induction time & date: :**

**Anaesthetic technique (*tick all that apply*)**

**⬜ General ⬜ Spinal ⬜ Epidural ⬜ Sedation / Local**

**⬜ Cancer Name………….**

**⬜ Procedure Name:**

**Cancer surgical procedure category (*single best answer*):**

**⬜UGI (Esophageal, stomach, and small bowel)**

**⬜Colorectal (Colon, rectum, anorectal condition, and appendix)**

**⬜ Endocrine (Breast, Thyroid, Parathyroid, and adrenal)**

**⬜ Obstetrics**

**⬜Gynecology**

**⬜ Urological surgery**

**⬜ Hepato-biliary**

**⬜ Vascular**

**⬜Thoracic**

**⬜ Cardiac**

**⬜Neurosurgery**

**⬜Orthopedics**

**⬜Plastic surgery**

**⬜Pediatrics surgery**

**⬜Ear, Nose, Throat, and maxillofacial**

**⬜Ophthalmology surgery**

**⬜Others**

**Severity of surgery ⬜ Minor ⬜ Intermediate ⬜ Major**

**Laparoscopic surgery ⬜ Y ⬜ N**

**Surgical checklist used (eg WHO checklist)⬜ Y ⬜ N**

**Critical care immediately after surgery ⬜ Y ⬜ N**

**Postoperative morbidity ⬜Yes ⬜No**

**If yes, Clavien–Dindo classification**

- **Grade 1**
- **Grade 2**
- **Grade IIIA**
- **Grade IIIB**
- **Grade IV A**
- **Grade IV B**
- **Grade V**

| **Grades** | **Definition** |
| --- | --- |
| **Grade I** | **Any deviation from the normal postoperative course without the need for pharmacological treatment or surgical, endoscopic and radiological interventions Allowed therapeutic regimens are: drugs as antiemetics, antipyretics, analgetics, diuretics and electrolytes and physiotherapy. This grade also includes wound infections opened at the bedside.** |
| **Grade II** | **Requiring pharmacological treatment with drugs other than such allowed for grade I complications. Blood transfusionsand total parenteral nutritionare also included.** |
| **Grade III** | **Requiring surgical, endoscopic or radiological intervention** |
| **- IIIa** | **Intervention not under general anesthesia** |
| **- IIIb** | **Intervention under general anesthesia** |
| **Grade IV** | **Life-threatening complication (including CNS complications)* requiring IC/ICU-management** |
| **- Iva** | **single organ dysfunction (including dialysis)** |
| **- IVb** | **Multiorgandysfunction** |
| **Grade V** | **Death of a patient** |

**Outcome after cancer surgery**

**Infection**

**Superficial surgical site Mild ⬜ Moderate ⬜ Severe ⬜ None ⬜**

**Deep surgical site Mild ⬜ Moderate ⬜ Severe ⬜ None ⬜**

**Body cavity Mild ⬜ Moderate ⬜ Severe ⬜ None ⬜**

**Pneumonia Mild ⬜ Moderate ⬜ Severe ⬜ None ⬜**

**Urinary tract Mild ⬜ Moderate ⬜ Severe ⬜ None ⬜**

**Bloodstream Mild ⬜ Moderate ⬜ Severe ⬜ None ⬜**

**Cardiovascular**

**Myocardial infarction Mild ⬜ Moderate ⬜ Severe ⬜ None ⬜**

**Arrhythmia Mild ⬜ Moderate ⬜ Severe ⬜ None ⬜**

**Pulmonary oedema Mild ⬜ Moderate ⬜ Severe ⬜ None ⬜**

**Pulmonary embolism Mild ⬜ Moderate ⬜ Severe ⬜ None ⬜**

**Stroke Mild ⬜ Moderate ⬜ Severe ⬜ None ⬜**

**Cardiac arrest Severe ⬜ None ⬜**

**Other**

**Gastro-intestinal bleed Mild ⬜ Moderate ⬜ Severe ⬜ None ⬜**

**Acute kidney injury Mild ⬜ Moderate ⬜ Severe ⬜ None ⬜**

**Post-operative bleed Moderate ⬜ Severe ⬜ None ⬜**

**ARDS Mild ⬜ Moderate ⬜ Severe ⬜ None ⬜**

**Anastomotic leak Mild ⬜ Moderate ⬜ Severe ⬜ None ⬜**

**Other Mild ⬜ Moderate ⬜ Severe ⬜ None ⬜**

**Treatment for post-operative complications:**

**Drug therapy, blood transfusion or parenteral nutrition ⬜ Y ⬜ N**

**Surgical or radiological procedure ⬜ Y ⬜ N**

**Critical care admission ⬜ Y ⬜ N**

**Hours in Post-Anaesthetic Care Unit after surgery**

h

h

**Days in critical care after surgery**

d

d

d

d

**What is the status of the patient at 7^th^ day?**

**⬜ Still in the hospital**

**⬜Discharged**

**⬜Refer to other hospital for further treatment**

**⬜Died**

**If discharged, Date of discharge………………….**

**Duration of hospital stay……………….**
